# Understanding the Catalytic Efficiency of Two Polyester Degrading Enzymes: An Experimental and Theoretical Investigation

**DOI:** 10.1021/acsomega.4c06528

**Published:** 2024-10-23

**Authors:** Matilda Clark, Konstantinos Tornesakis, Gerhard König, Michael Zahn, Bruce R. Lichtenstein, Andrew R. Pickford, Paul A. Cox

**Affiliations:** † Centre for Enzyme Innovation, University of Portsmouth, St Michael’s Building, Portsmouth PO1 2DT, U.K.; ‡ School of Medicine, Pharmacy and Biomedical Sciences, University of Portsmouth, St Michael’s Building, Portsmouth PO1 2DT, U.K.

## Abstract

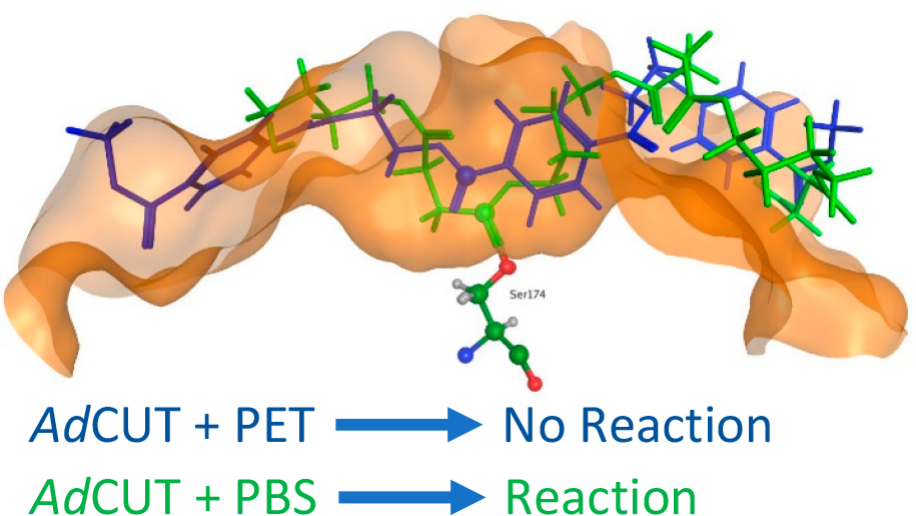

The discovery of novel plastic degrading enzymes commonly relies on comparing features of the primary sequence to those of known plastic degrading enzymes. However, this approach cannot always guarantee success. This is exemplified by the different degradation rates of the two polymers poly(ethylene terephthalate) (PET) and polybutylene succinate (PBS) by two hydrolases: *Is*PETase from *Ideonella sakaiensis* and *Ad*Cut from *Acidovorax delafieldii*. Despite the enzymes showing a very high sequence identity of 82%, *Is*PETase shows significant hydrolysis activity for both polymers, whereas *Ad*Cut only shows significant hydrolysis activity for PBS. By solving the structure of *Ad*Cut using X-ray crystallography, and using this as the basis for computer simulations, comparisons are made between the differences in the calculated binding geometries and the catalytic results obtained from biochemical experiments. The results reveal that the low activity of *Ad*Cut toward PET can be explained by the low sampling of the productive conformation observed in the simulations. While the active site serine in *Is*PETase can closely encounter the PET carbonyl carbon, in *Ad*Cut it cannot: a feature that can be attributed to the shape of the catalytic binding pocket. These results yield an important insight into the design requirements for novel plastic degrading enzymes, as well as showing that computational methods can be used as a valuable tool in understanding the molecular basis for different hydrolysis activities in homologous polyesterase enzymes.

## Introduction

Plastics have become widely used due to their low cost and high durability. However, this property has led to their accumulation in terrestrial and marine environments, which has severe ecological consequences.^1^ Despite this, the use of plastics is still increasing without a concomitant increase in the amount of plastic recycling.^2^ One possible solution to this problem is the biocatalytic recycling of plastic polymers by hydrolase enzymes into monomer units. This allows the plastics to be recycled into products with the same qualities as those produced from fossil fuel sources. However, recycling on an industrial scale requires enzymes with high activity, selectivity, stability and solubility, among many other requirements. Therefore, investigations into the requirements for activity could help to accelerate the discovery of new enzymes with beneficial properties.

To study the different selectivities of hydrolases, two enzymes, *Is*PETase and *Ad*Cut, were investigated. *Is*PETase was isolated from the bacterium *Ideonella Sakaiensis*, an organism able to use poly(ethylene terephthalate) (PET) as a major carbon and energy source.^3^ PET is the most commonly used polyester due to its durability and low cost of production.^4^*Is*PETase degrades PET into mono(2-hydroxyethyl)terephthalic acid (MHET), terephthalic acid (TPA), and bis(2-hydroxyethyl) terephthalate (BHET).^5^ The second enzyme, *Ad*Cut, is found in the bacteria *Acidovorax delafieldii*, which is able to grow on poly(butylene succinate) (PBS) as its sole carbon and energy source.^6,7^ In a recent study, *Ad*Cut was investigated for PET degrading activity due to the similarity of its primary sequence to that of *Is*PETase but was found to have very poor activity against PET.^8^ However, the structure and reason behind the lack of activity were not investigated. *Is*PETase and *Ad*Cut are highly homologous (82% sequence identity and 89% sequence similarity). Therefore, these two enzymes are interesting targets to study in order to understand the low activity of *Ad*Cut against PET since this could help us to provide more informed design and search criteria for identifying novel plastic degrading hydrolases.

*Is*PETase follows the classical mechanism of cutinases.^1,9^ In the acylation stage, a Ser residue performs a nucleophilic attack on the polymer’s ester bond. An oxyanion hole is formed by Tyr and Met residues, which polarize the carbonyl oxygen of the ester bond and stabilize an acyl–enzyme reaction intermediate. In the deacylation stage, the acyl reaction intermediate is resolved in a nucleophilic attack by a water molecule, restoring the enzyme to its original state. Given the sequence identity and the presence of the same catalytic triad, *Ad*Cut most likely uses the same reaction mechanism. There are several conditions that must be fulfilled before the reaction can occur: (a) the substrate must bind in the vicinity of the catalytic triad, (b) the enzyme–substrate complex must assume the productive conformation, and (c) the catalytic reaction proceeds through the intermediate state(s) to produce the product.

In this study, a combined theoretical and experimental approach was applied to understand the specificity of the plastic degrading enzymes *Is*PETase and *Ad*Cut toward PET and PBS polymers. Biochemical experiments were used to investigate the activity and binding of the two hydrolases on PET, PBS, and BHET (a diester mimic of the PET polymer), and binding of the enzymes to solid substrates was measured. The crystal structure of *Ad*Cut was solved, which established the positioning of the catalytic triad in the enzyme. Molecular docking was then employed to model enzyme–substrate complexes in order to determine the energy and geometry of the substrate at the active site. Finally, the structural flexibility of the complex was explored with molecular dynamics simulations. These simulations were used to model the conformation and stability of the enzyme–substrate complex and reveal the likelihood of encountering the productive conformation as a function of time. This has allowed for a deeper understanding of the differences between homologous enzymes and the properties required when searching for industrially relevant biocatalysts.

## Methods

### Materials

PET was supplied by Goodfellow as an amorphous film. PBS was supplied as granules from Shijiazhuang Tuya Technology Co., Ltd. Micronized amorphous powder was produced from the PET film and PBS pellets by cryomilling in a ZM200 centrifugal mill (Retsch) using a 0.12 mm sieve. After air drying, the particle size and shape distributions in the PET powder were assessed by dynamic image analysis using a CAMSIZER X2 (Microtrac MRB) with an X-Fall module. Thereafter, an approximation of the powder surface area was calculated from the derived distributions of particle cross-sectional area and aspect ratio (Figure S1).

### Expression and Purification of *Ad*Cut and *Is*PETase

The *Ad*Cut and *Is*PETase genes were purchased from Twist Bioscience cloned into the PET-21b(+) vector. Nucleotide and protein sequences can be found in the Supporting Information (Tables S1 and S2). The proteins were expressed and purified according to an established method.^8^

### Crystallization and Structure Determination of *Ad*Cut

For crystallization, the protein was concentrated to 4.6 mg/mL and sitting drop crystallization trials were set up with a mosquito crystallization robot (sptlabtech) using SWISSCI 3-lens low profile crystallization plates. *Ad*Cut crystals appeared in condition A3 of the JCSG-plus screen from Molecular Dimensions (20% PEG 3350, 0.2 M ammonium citrate dibasic).

The crystal was cryoprotected with 20% glycerol in the crystallization solution and flash-frozen into liquid nitrogen. Diffraction data were collected at Diamond Light Source (Didcot, UK) and automatically processed using STARANISO^10^ on ISPyB. The structure was solved by molecular replacement with MOLREP^11^ using an AlphaFold structure prediction.^12^ Model building was performed in Coot,^13^ and the structure was refined with REFMAC5.^14^

### Biochemical Assays

The phenol red assay by Lusty Beech et al.^15^ was used to measure the enzymatic hydrolysis of BHET. As BHET is a reasonably soluble substrate, the phenol red assay could be used to continuously measure the progress of the reaction. Phenol red indicator at a concentration of 0.1 mM was used to measure the acidification of the reaction at 550 nm as ester bonds were cleaved.^15^ To measure *Ad*Cut activity against BHET, 5 μM enzyme was added to 2 mM BHET in 5 mM HEPES pH8 in a volume of 150 μL. To measure *Is*PETase against BHET, the conditions were the same except for using 10 nM *Is*PETase. The Infinite MNano+ (Tecan) was used to read the absorbance at 550 nm every 5 min for 2 h at room temperature.

For the comparison of *Is*PETase and *Ad*Cut activities against different solid substrates, high-performance liquid chromatography (HPLC) was used. This allowed for more sensitive measurements and confirmation of the molecular breakdown products. To determine the reaction rate against these solid substrates, 10 mg of PET or PBS powder was incubated with 1 μM enzyme. The solution was incubated at 30 °C, shaking at 600 rpm. To stop the reaction, the solution was incubated at 90 °C for 10 min and centrifuged at 21 130*g* for 10 min to remove the residual powder.

To test the activity of *Ad*Cut in the presence of PET, the same method was used with 1 μM *Ad*Cut with 4 mg of PBS powder. A second experiment was set up with 4 mg of PBS powder as well as 4 mg of amorphous PET powder. The reaction volume was 100 μL, and the reaction was incubated at 30 °C, shaking at 600 rpm.

To measure the breakdown of PBS, the released succinic acid was quantified using an Agilent 1200 HPLC system equipped with a diode array detector at a wavelength of 210 nm and using a Phenomenex Luna C18 column, 5 μm, 4.6 mm × 150 mm. The column temperature was maintained at 25 °C, and the mobile phase used to separate the analytes of interest was composed of 20 mM phosphoric acid in water (A) and 100% acetonitrile (B). The separation was carried out using a constant flow rate of 0.6 mL/min with a linear gradient from 5 to 95% acetonitrile over 15 min followed by 5 min of 95% acetonitrile. A calibration curve was performed with succinic acid (Sigma-Aldrich) at concentrations between 0.78 and 100 mM and analyzed with the same conditions. To measure the breakdown of PET, the same method as described previously was used.^16^

### Binding Assays

To determine equilibrium dissociation constant (*K*_D_) values for PET binding, a previously described method with minor modifications was used.^17^ For each enzyme construct, a fixed mass loading of PET or PBS (*L*_PET/PBS_ = 66.7 g/L) in a final volume of 150 μL was incubated with 0.313–10 μM enzyme overnight at 4 °C in 1.5 mL microcentrifuge tubes. The total enzyme concentration prior to substrate addition (*E*_total_) and, following each incubation, the free-enzyme concentration (*E*_free_) were determined using a Micro BCA kit (Pierce). To account for possible variations in the reduction of Cu^2+^, a standard curve was generated for each enzyme over a concentration range of 9.8 nM–10 μM. Thereafter, the substrate coverage Γ was calculated as Γ = *E*_total_*E*_free_/*L*_PET/PBS_ and plotted as a function of *E*_free_. In order to obtain the *K*_D_, the data was fitted to the equation describing a Langmuir adsorption isotherm
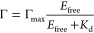
1where Γ max is the substrate coverage at the surface saturation.

### Molecular Docking

The program Molecular Operating Environment (MOE)^18^ was used to identify minimum energy locations within *Ad*Cut and *Is*PETase for trimers of the PBS and PET polymers, as well as for a single molecule of BHET. Docking was performed using the Dock module within MOE. The coordinates determined in this study were used for *Ad*Cut, and the coordinates from the Protein Data Bank (code: 6EQE) were used for *Is*PETase. Initial placement of the oligomer chains was performed using the Triangle Matcher method, and these positions were scored using the London dG approach. The top 200 poses were then energy-minimized using the Amber10-EHT force field and charges. The minimization was terminated when the RMS gradient fell below 0.01. Charges for small molecules, BHET, PBS, and PET, were calculated using AM1-BCC, as specified for use with this force field. An induced fit model was used with the side chains tethered with a default weight factor of 10. The binding free energy of the trimer was estimated by using the GBVI/WSA dG function. The minimum energy locations for PBS and PET trimers were subsequently used as starting points for the molecular dynamics calculations.

### Molecular Dynamics Simulations

Molecular dynamics simulations of *Ad*Cut and *Is*PETase with docked PBS and PET trimers were performed with the AMBER20 package.^19^ The GAFF force field^20^ with AM1-BCC charges^21^ was used for the ligands, while the FF19SB force field^22^ was employed for the proteins. The structures of the proteins were prepared with H++.^23^ The proteins were surrounded with 13–14 Å of TIP3P water^24^ and 0.1 M of sodium chloride in a cubic box. Langevin dynamics were performed with a time step of 2 fs using SHAKE,^25^ a friction coefficient of 1 ps^–1^, and a temperature of 300 K. The pressure was maintained at 1 atm with a Monte Carlo barostat.^26^ The cutoff was 12 Å using force-switching^27^ with a switching region of 2 Å. Electrostatic interactions were treated with the particle mesh Ewald method.^28^ After 5000 steps of energy minimization with the steepest descent, the structure was heated to 300 K for 0.125 ns, followed by 10 ns of equilibration and 100 ns of production. The trajectories were processed with cpptraj^29^ to generate histograms of the distances between the main reaction partners. Since the trimers exhibit multiple bonds that can be broken, only the closest reaction partner in each simulation was considered for the histograms.

## Results

### Structure of *Ad*Cut

*Ad*Cut was expressed and purified, and the structure was solved to a 1.5 Å resolution (Figure S1 and Table S3). The coordinates have been deposited in the Protein Data Bank under accession code 8C65. Solving the structure of *Ad*Cut enabled the verification of the positioning of the functionally active catalytic triad, as well as providing an experimental structure for modeling substrate interactions. As expected from the high degree of sequence identity with *Is*PETase, the RMSD value between both structures is low, 0.41 Å (262 aligned residues). Consequently, the canonical catalytic triad of *Ad*Cut (residues S174, D220, and H251) aligns very well with that of *Is*PETase (residues S160, D206, and H237), with the residues surrounding the catalytic pocket very similar for both enzymes (see Table 1).

**Table 1:** List of the Residues Surrounding the Binding Pocket in *Ad*Cut and *Is*PETase

enzyme	residues surrounding the binding pocket
*Ad*Cut (**PDB:8C65**)	GLY100, PHE101, THR102, ALA103, TRP173, SER174, MET175, TRP199, ILE222, HIS215
*Is*PETase (**PDB:6EQE**)	GLY86, ALA87, ALA89, TRP159, SER160, MET161, TRP185, ILE208, ALA209, HIS237

**Figure 1:**
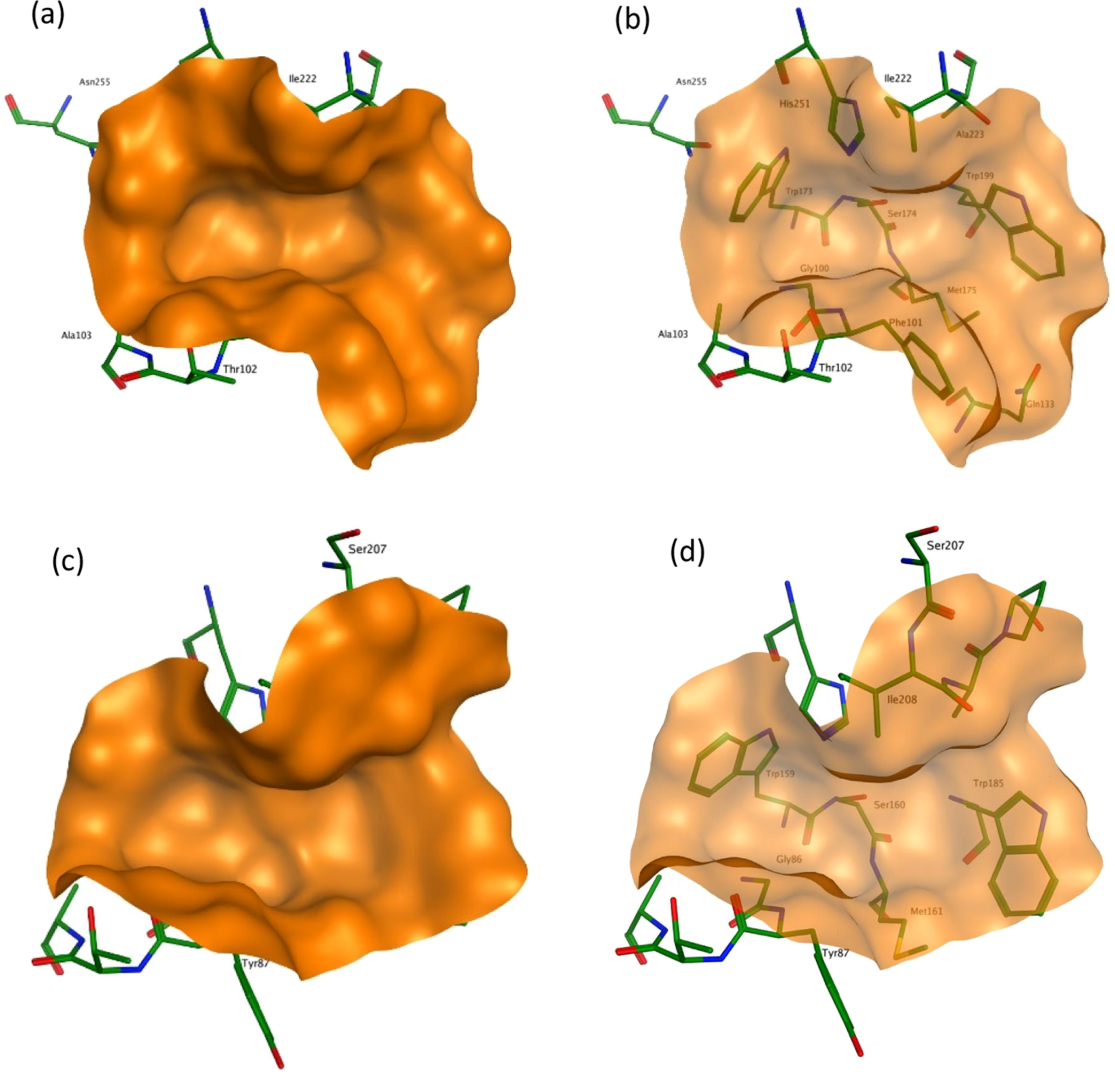
Solid representations of the binding cavity in *Ad*Cut (PDB: 8C65) (a) and *Is*PETase (PDB: 6EQE) (c). Transparent views of the binding cavity in *Ad*Cut (b) and *Is*PETase (d) allow the arrangement of the residues to be seen.

However, despite these similarities, it is interesting to note subtle differences in the shapes of the two binding pockets (Figure 1). The cavity in *Is*PETase is broader, slightly longer, and, toward the right-hand side, is shallower when compared with the cavity in *Ad*Cut.

### Characterization of *Is*PETase and *Ad*Cut Activities against BHET, PBS, and PET

**Figure 2:**
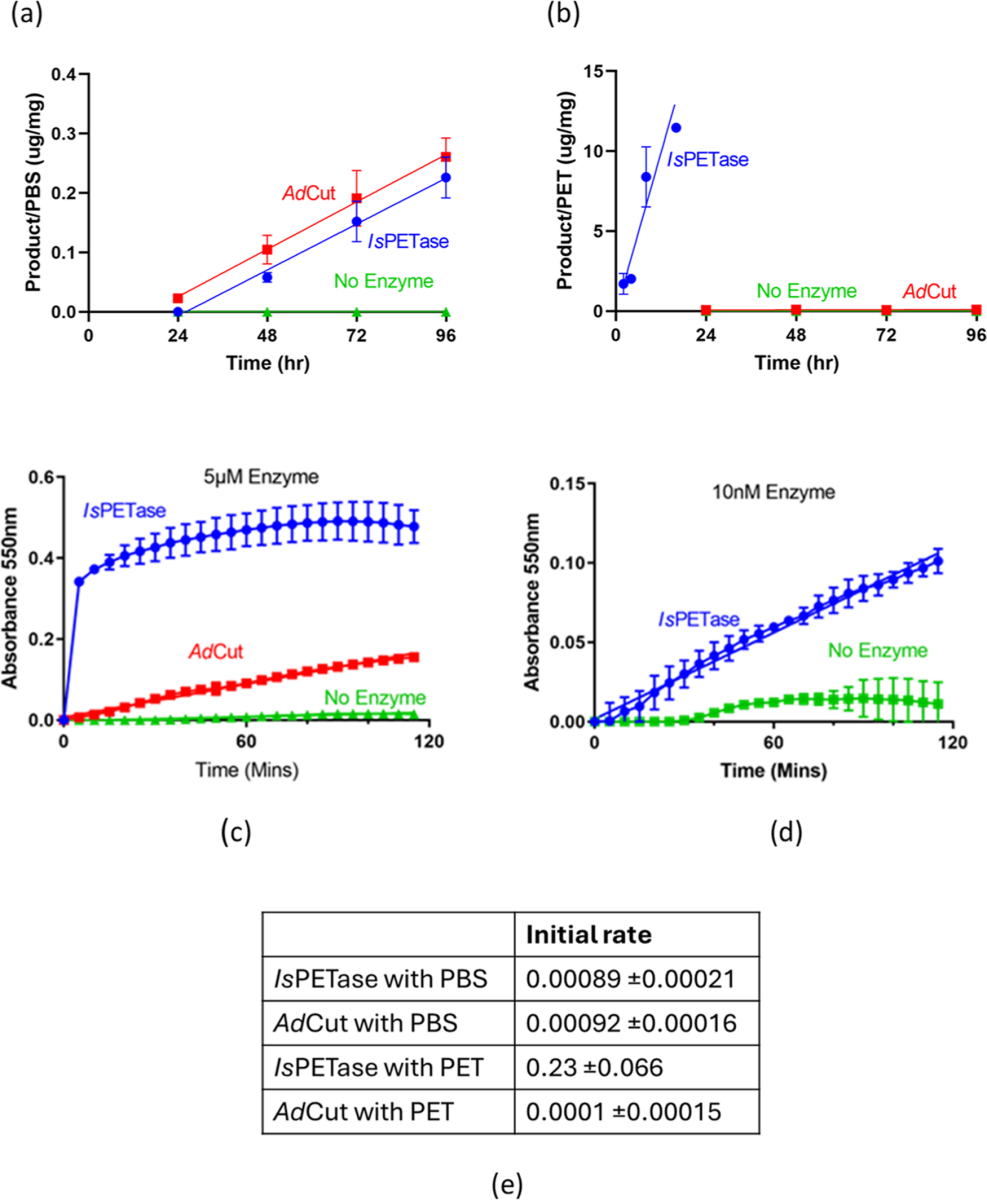
Quantification of *Ad*Cut and *Is*PETase activities against BHET, PBS, and PET. Panels (a, b) show HPLC measurements of product formation for PBS and PET with *Ad*Cut and *Is*PETase. Panel (a) shows succinic acid formation per mg PBS in the presence of *Ad*Cut and *Is*PETase. Panel (b) shows product formation (TPA, MHET, and BHET) per mg of a PET solution in the presence of *Ad*Cut and *Is*PETase. Panels (c, d) show the change in the absorbance of phenol red with BHET in the presence of *Ad*Cut and *Is*PETase. In panel (c), the experiment was carried out with 5 μM enzyme. *Is*PETase showed a very fast rate that could not accurately be quantified and so was repeated with 10 nM enzyme as shown in panel (d). Data are the means ± SD of two repeats. (e) Initial rates calculated from the gradients in ng of product formed per milligram of plastic per second.

The rates of enzymatic activity of *Ad*Cut and *Is*PETase against both PBS and PET were determined by HPLC experiments (Figure 2). Figure 2a shows the product formation of succinic acid when PBS is incubated in the presence of *Ad*Cut and *Is*PETase. The control represents PBS-added enzymes. This shows that both enzymes can catalyze this reaction with similar initial rates.

For PET (Figure 2b), product formation (TPA, MHET, and BHET) shows that only *Is*PETase is able to catalyze this reaction, while *Ad*Cut did not cause any significant PET degradation above the negative control.

The phenol red assay was used to measure the amount of BHET cleaved by the two enzymes. The change in the absorbance of the phenol red indicator indicates a change in pH due to the release of acidic products. When tested in the phenol red assay, *Ad*Cut was seen to have poor activity against BHET in comparison to *Is*PETase as shown in Figure 2c. The assay needed to be carried out at a lower concentration of PETase in order to calculate the rate of the reaction. Therefore, the experiment was repeated with a 10 nM enzyme (Figure 2d). The rate of reaction shows that the activity of *Is*PETase on BHET is approximately 323 times faster than that of *Ad*Cut. The comparatively low activity of *Ad*Cut on BHET, which is a small molecule, suggests that the decrease in PET hydrolase activity is caused at the active site rather than the inability of the extended chain to bind to areas further away.

**Figure 3:**
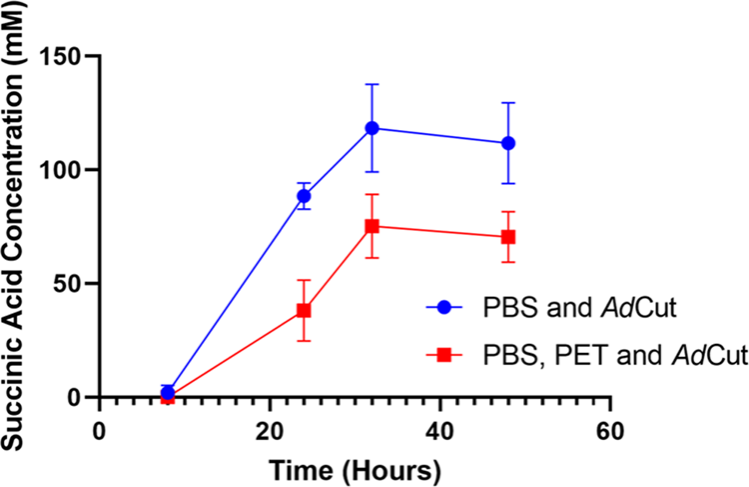
Competitive inhibition of PBS degradation by PET in *Ad*Cut was measured by HPLC. The conversion of PBS into succinic acid is reduced by the addition of PET, which indicates competitive inhibition due to the binding of PET to the same binding site as that of *Ad*Cut. Data are the means ± SD of three repeats.

The rate of succinic acid production from PBS by *Ad*Cut in the presence of PET was measured using HPLC as shown in Figure 3. When PET was added to a reaction mixture, it caused a significant decrease in the amount of succinic acid being produced. The rate of production of succinic acid was slower and plateaued at a lower level compared to PBS and *Ad*Cut alone. This suggests that PET is binding to *Ad*Cut active sites and competitively inhibits the turnover of PBS. When recycling a mixture of plastics on an industrial scale, this change of conversion rates can be the difference between a process that is economically viable and one that is not.

### Binding Assays

**Figure 4:**
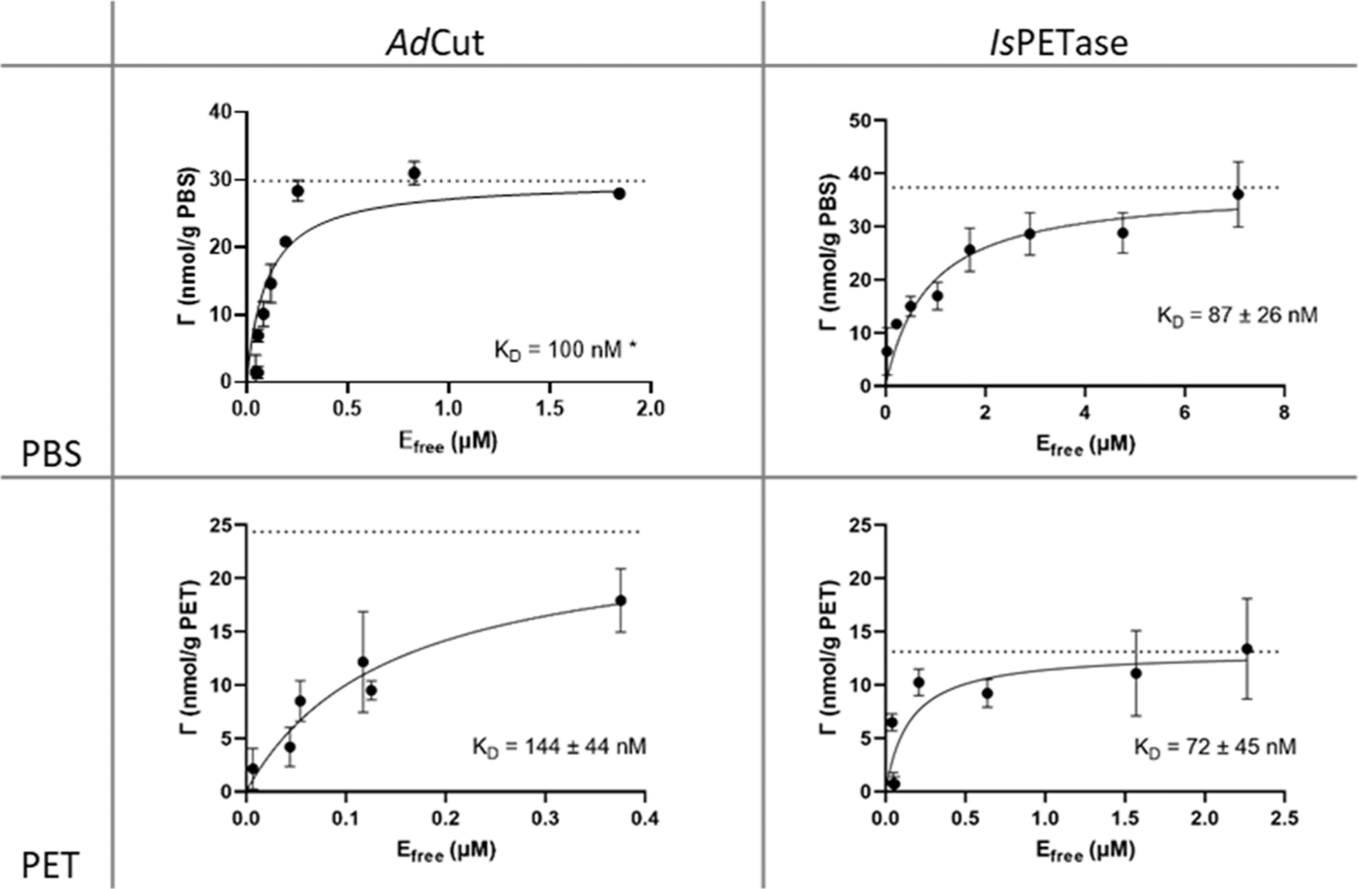
Binding of *Ad*Cut and *Is*PETase to PBS and PET powders. Based on the substrate coverage (Γ) in response to the free-enzyme concentration (*E*_free_), the dissociation constants (*K*_D_ in the insets) were calculated according to eq 1. The nanomolar *K*_D_ shows that both enzymes exhibit a strong binding affinity to both substrates. All data are the means ± SD of three replicates. *R*^2^ values for the fitting of this data are shown in Table S4. The asterisk indicates that *K*_D_ was constrained to improve data fitting when the maximum binding was unclear. Data are the means ± SD of three repeats.

The binding of PBS and PET to the enzymes was measured based on Langmuir isotherms (Figure 4). These assays confirm that both enzymes have the ability to bind the two polymers.

The derived equilibrium dissociation constant (*K*_D_) values reveal that *Ad*Cut has a similar affinity for PBS and PET, while *Is*PETase has a higher binding affinity for PET. All *K*_D_ values are in the nanomolar range, which indicates strong binding. These results confirm the ability of *Ad*Cut to bind the PET polymer despite observing no breakdown of the polymer in biochemical assays.

### Docking Calculations

To determine whether the substrates are capable of binding to the catalytic center, molecular docking calculations were performed. The calculated binding free energies for PET and PBS trimers and BHET to the *Is*PETase and *Ad*Cut enzymes are shown in Table 2. The negative binding free energies show that all enzyme–substrate pairs form stable complexes. The binding affinities for PBS and PET are both higher in *Is*PETase than in *Ad*Cut, in agreement with the experimental results. The observed binding energies for PET are lower than PBS in both *Is*PETase and *Ad*Cut; however, this may be attributed to the fact that the PBS trimer contains more atoms than the PET trimer. Since both PET and PBS bind strongly to both enzymes, this suggests that any differences in substrate specificity must arise from later stages of the reaction mechanism.

**Table 2:** Lowest Binding Free Energies (kcal mol^–1^) for the Enzyme–Substrate Complexes

enzyme/substrate	binding energy/kcal mol^–1^
*Is*PETase–BHET	–5.69
*Is*PETase–PET	–7.97
*Is*PETase–PBS	–8.12
*Ad*Cut–BHET	–5.49
*Ad*Cut–PET	–7.52
*Ad*Cut–PBS	–7.61

**Figure 5:**
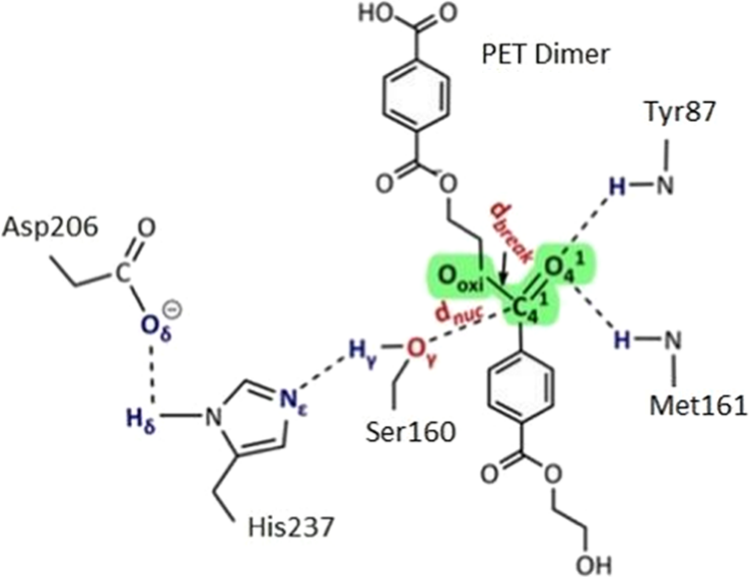
Rate-determining acylation step in the reaction mechanism of *Is*PETase. The *H*_γ_ of Ser is first transferred to *N*_ϵ_ of His, followed by a nucleophilic attack of *O*_γ_ on *C*_C=O_ of the substrate. The reported average distances of *H*_γ_–*N*_ϵ_ and *O*_γ_–*C*_C=O_ in the productive conformation are 1.8 and 3.3 Å, respectively. Adapted with permission from ref (1). Copyright 2019 American Chemical Society.

The rate-determining step of the reaction mechanism of *Is*PETase involves the acylation of the substrate.^1^ The acylation starts with a proton transfer of *H*_γ_ from Ser160 to His237, followed by a nucleophilic attack of Ser160 on the substrate (Figure 5). To proceed with the reaction, the catalytic residues and the substrate must be in the productive conformation, which is characterized by distances between *H*_γ_–*N*_ϵ_ and *O*_γ_–*C*_C=O_ of about 1.8 and 3.3 Å, respectively.

To estimate whether different enzyme–substrate pairs are equally capable of forming the productive conformation, the *O*_γ_–*C*_C=O_ distances of the docked structures were evaluated. Analysis of the *O*_γ_–*C*__C=O__ distance for BHET in the two enzymes is particularly interesting. BHET binds more strongly to *Is*PETase (−5.69 kcal mol^–1^) than to *Ad*Cut (−5.49 kcal mol^–1^). The *O*_γ_–*C*_C=O_ distance in *Is*PETase (3.07 Å) is significantly less than that observed for *Ad*Cut (3.74 Å), with the distance in *Ad*Cut being significantly longer than the value of 3.3 Å associated with the productive conformation.

**Figure 6:**
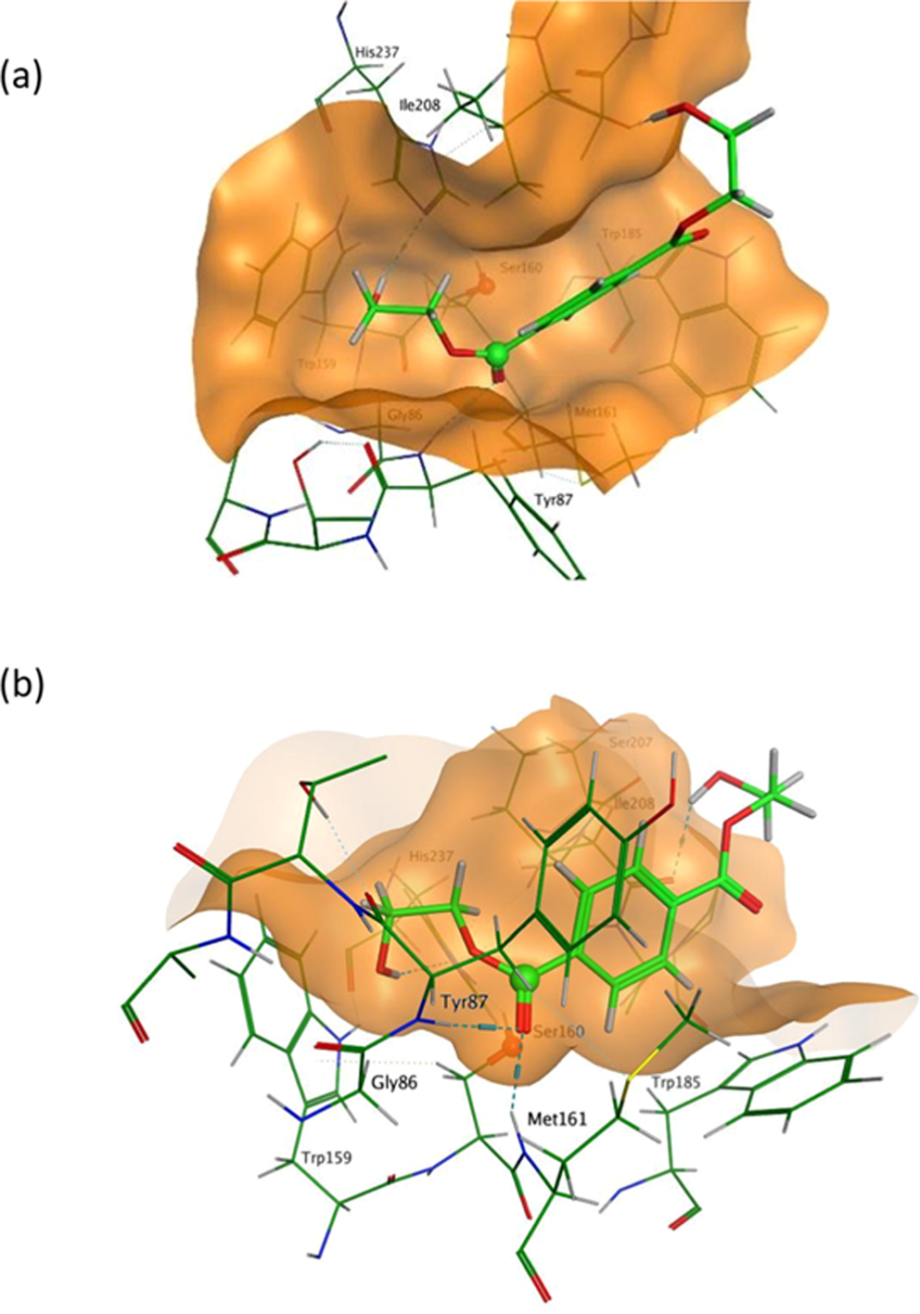
Minimum energy binding configuration for BHET in *Is*PETase. (a) The BHET molecule is a good fit inside the catalytic pocket (shown by the orange surface). (b) The position of the BHET molecule relative to the surrounding residues shows that the *C*_C=O_ atom (green sphere) on the substrate is placed in the proximity (3.07 Å) of the O_γ_ atom (red sphere) on Ser160.

Figure 6 shows the minimum energy configuration for BHET in the active site of *Is*PETase. It can be seen that the shape of the binding pocket is such that it favors the location of the carbonyl group and the adjacent ring at positions that match well with the contours of the pocket. In this location, *C*_C=O_ on the BHET molecule is guided to a position that is in close proximity (3.07 Å) to the *O*_γ_ atom on Ser160. Furthermore, it can be seen that the H-bond formed between the *O*__C=O__ on BHET and Tyr87 and Met161 helps to position the BHET molecule for nucleophilic attack. In essence, the shape of the binding pocket is very well matched to that of the BHET molecule, ensuring that the catalytic reaction can begin to proceed.

**Figure 7:**
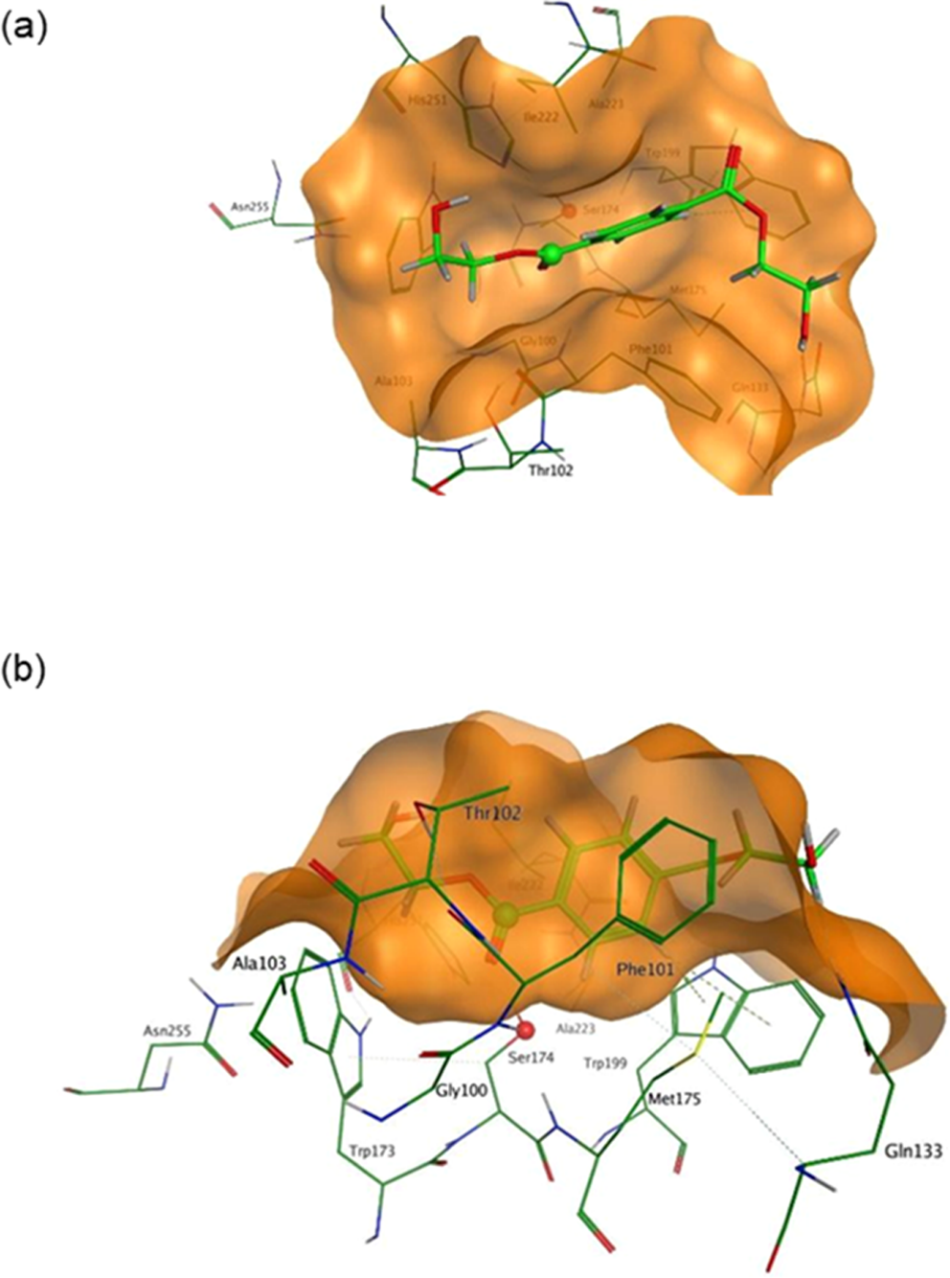
Minimum energy binding configuration for BHET in *Ad*Cut. (a) Fit of the BHET molecule inside the catalytic pocket (shown by the orange surface). (b) The position of the BHET molecule relative to the surrounding residues shows that the *C*__C=O__ atom on the substrate is placed further away (3.74 Å) from the *O*_γ_ atom on Ser174 than observed in *Is*PETase.

In contrast, the binding of BHET to *Ad*Cut does not produce such a favorable geometry (Figure 7). In this case, the observed *O*_γ_–*C*_C=O_ distance is much less favorable (3.74 Å) and exceeds the *O*_γ_–*C*__C=O__ distance typically expected for the productive conformation. In this case, the shape of the pocket is such that accommodating the *C*_C=O_ atom in the vicinity of Ser174 necessitates the inclusion of a rigid aryl ring within the binding cavity. Space in the pocket is limited, thus keeping the *C*_C=O_ atom further away from *O*_γ_ on the Ser174 residue, which is located centrally in the cavity. The restricted space surrounding the catalytic Ser174 residue in *Ad*Cut appears to be playing a key role in hindering the reaction.

**Figure 8:**
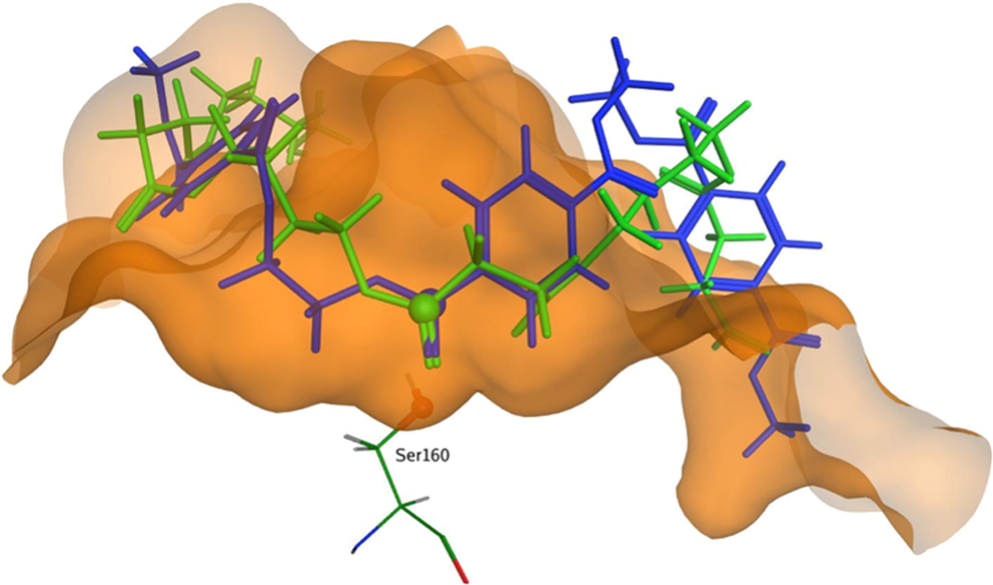
Minimum energy positions of PET (blue) and PBS (green) docked into *Is*PETase. The C_C=O_ position for both trimers is in close proximity, and the distances to the *O*_γ_ atom on Ser160 are both expected to lead to reaction (3.17 and 3.32 Å for PET and PBS, respectively).

The PET trimer docks into the *Is*PETase binding pocket in an analogous way to that observed for BHET. The −(C=O)–(C_6_H_4_)–(C=O)– moiety that PET shares with BHET has the same good “shape-match” between this part of the polymer and the binding pocket. This leads to a similar *O*_γ_–*C*_C=O_ distance (3.17 Å) to the one observed for BHET, a suitable distance for the initiation of the catalytic reaction. The minimum energy position for the PBS trimer in the *Is*PETase binding pocket has a *O*_γ_–*C*_C=O_ distance of 3.32 Å. Again, this is consistent with a distance that would allow the reaction to proceed. Figure 8 shows the PBS and PET dimers docked into *Is*PETase at their lowest energy positions.

**Figure 9:**
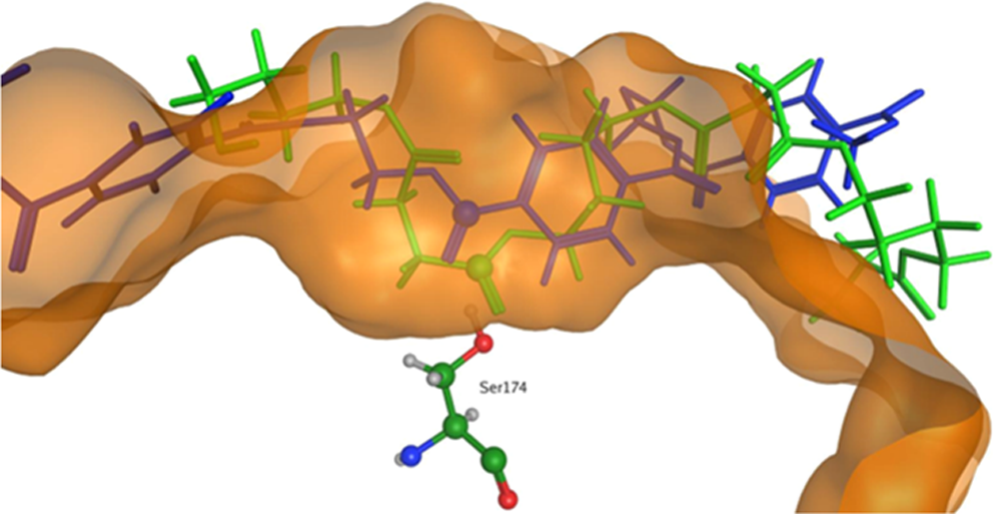
The *O*_γ_–*C*_C=O_ distances observed for the lowest energy position for PET (blue) and PBS (green) docked into *Ad*Cut are significantly different. The lowest value for PBS is 2.98 Å, whereas that observed for PET is 3.94 Å.

The minimum energy configurations for PBS and PET trimers in *Ad*Cut have very different *O*_γ_–*C*__C=O__ distances. For PBS, the distance is 2.98 Å, whereas for PET, it is much longer, 3.94 Å. In the region of the *Ad*Cut binding pocket, PET binds in a way similar to that observed for BHET, resulting in a *O*_γ_–*C*_C=O_ distance that is potentially too long for the reaction to proceed. Thus, the reaction is “shape-selective” toward PBS compared with PET because of the unfavorable geometric match between PET and the binding cavity in *Ad*Cut. The greater flexibility of the PBS polymer allows it to “fit” inside the binding cavity and achieve *O*_γ_–*C*_C=O_ distances required for the reaction. Figure 9 shows the docking positions for PBS and PET trimers at their minimum energy positions.

It is interesting to note that the docking results are consistent with the experimental observation that competitive inhibition was displayed by PET in the presence of PBS for the catalytic reaction using *Ad*Cut as the enzyme. Both polymers bind strongly to the active site, but PET does not react due to the inability of the substrate to adopt distances that are consistent with the required productive conformation. This reduces the number of available catalytic sites available for the breakdown of PBS, thereby decreasing the reaction rate.

In order to further explore the relative probabilities of the productive conformation for the two polymers inside the enzymes, molecular dynamics calculations were also performed. These calculations have the added advantage that they allow the investigation of the time evolution of the system at a specified temperature.

### Molecular Dynamics Simulations

**Figure 10:**
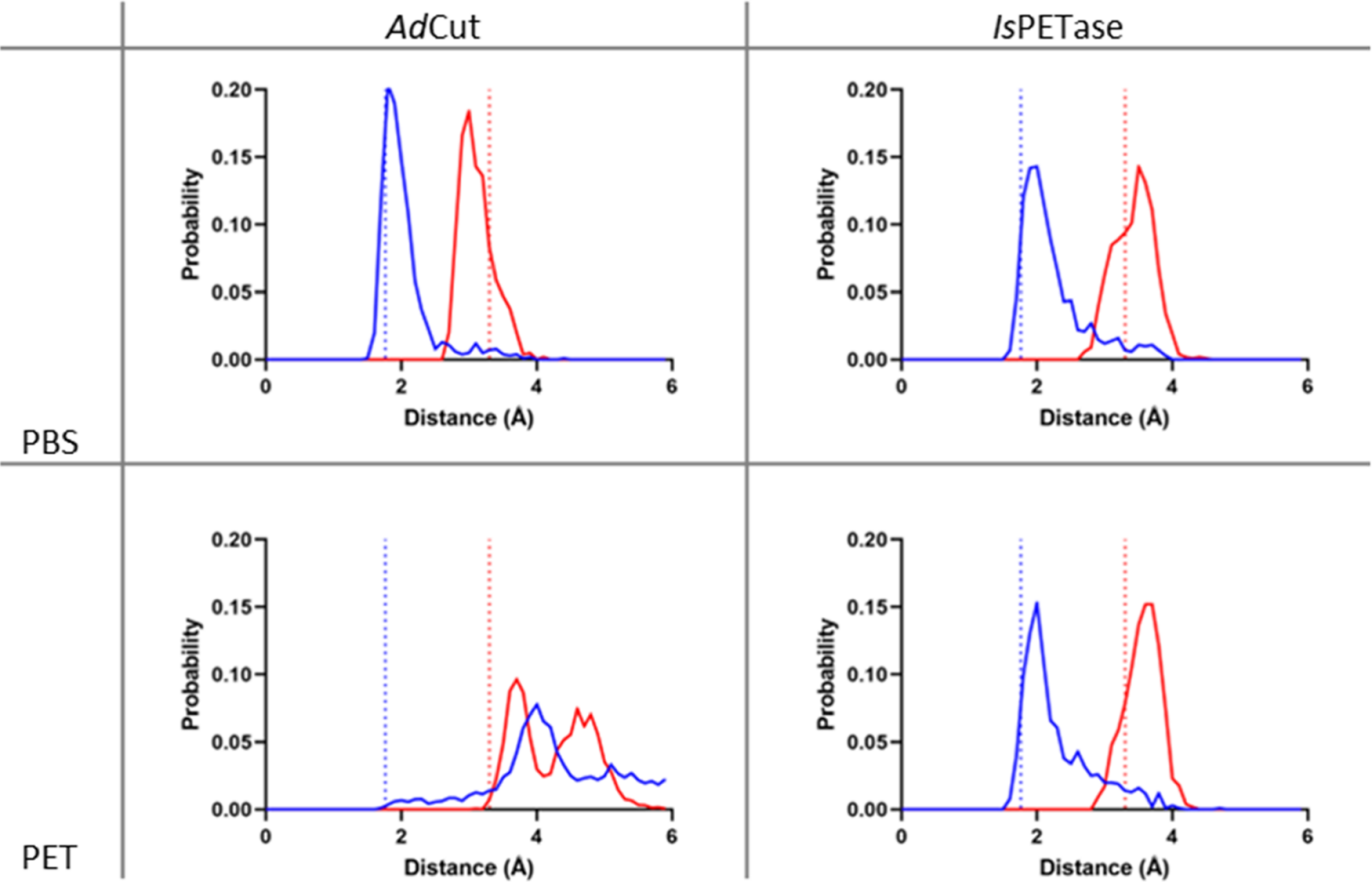
Probability distributions of the distances between *H*_γ_–*N*_ϵ_ (blue) and *O*_γ_–*C*_C=O_ (red) from molecular dynamics simulations. The blue dotted line on the *x*-axis indicates the average *H*_γ_–*N*_ϵ_ distance of the productive conformation, while the red dotted line indicates the average *O*_γ_–*C*_C=O_ distance of the productive conformation.^1^ Except for *Ad*Cut with PET, all enzyme–ligand combinations can adopt a productive conformation for the acylation reaction.

Molecular dynamics simulations of *Is*PETase and *Ad*Cut with PET and PBS trimers were performed for 100 ns of simulation time. The resulting histograms of the *H*_γ_–*N*_ϵ_ (blue) and *O*_γ_–*C*_C=O_ (red) distances are shown in Figure 10.

The presence of close *O*_γ_–*C*_C=O_ distances (red peaks) in all four subplots of Figure 10 demonstrates that both enzymes are able to bind both substrates. *Ad*Cut with PBS exhibits a peak of the *H*_γ_–*N*_ϵ_ distance at about 1.9 Å and a peak of the *O*_γ_–*C*_C=O_ distance at about 3.0 Å. Thus, the substrate can assume the same productive conformation as reported for *Is*PETase and PET in ref (1) (indicated by dashed lines). Likewise, the trajectory of *Is*PETase with PBS assumes a productive conformation. While the peak of the *O*_γ_–*C*_C=O_ distance distribution of PBS in *Ad*Cut appears to be slightly higher than with *Is*PETase, the observed difference is most likely not significant as it is within the “thermal noise” of the simulation (an energy difference of ca. 0.2*k*_*B*_*T*). Thus, PBS can assume the productive conformation in both *Is*PETase and *Ad*Cut, allowing the reaction to proceed.

On the other hand, the trajectory of *Ad*Cut with PET shows no significant probability of the productive conformation. The interaction with the carbonyl oxygen shows the peak of the *O*_γ_–*C*_C=O_ distance at much longer distances, hindering the nucleophilic attack on the substrate, in agreement with the results observed from the docking studies. In addition, the dynamics calculations reveal that the *H*_γ_ of the catalytic serine points toward the *N*_ϵ_ of the histidine in the unbound state, but with PET bound, its conformation changes, with the *H*_γ_ of the catalytic serine forming a hydrogen bond with the carbonyl oxygen of PET instead. Therefore, the peak of the *H*_γ_–*N*_ϵ_ probability distribution is shifted from 1.8 to around 4.0 Å, which would hinder the proton transfer reaction between the serine and the histidine. This suggests that, while *Ad*Cut does bind PET, it is not correctly positioned within the active site for the hydrolysis reaction to proceed. In contrast, *Is*PETase with PET is able to adopt the right distances for productive conformation, which allows the reaction to proceed.

## Discussion

The two highly homologous hydrolases *Ad*Cut and *Is*PETase share the same fold and catalytic triad but exhibit different substrate specificities. As shown in the binding assays and competition experiments, both enzymes exhibit high binding affinity for PET and PBS, but only *Is*PETase can significantly degrade PET. While the productive conformation for the reaction is formed with high probability in *Is*PETase for both substrates, in *Ad*Cut this is only the case for PBS showing that the binding of the substrate to the enzyme is not the only requirement for catalytic activity. For *Ad*Cut with PET, the nucleophilic attack is sterically hindered with the *O*_γ_–*C*_C=O_ unable to adopt the necessary distance expected for the reaction. The lack of *Ad*Cut activity on a nonpolymer, such as BHET, confirms that the steric hindrance is occurring close to the active site and again longer *O*_γ_–*C*_C=O_ distances are observed in the docking calculations, providing an explanation for the experimental observations. Thus, it can be concluded that the experimentally observed substrate specificities can be rationalized by using a combination of docking calculations and molecular dynamics simulations. This work highlights the important relationship between the geometry of the binding site and the substrate, helping to guide the search for novel and effective new plastic degrading enzymes.

The computational approach outlined in this work could be used in the future as an additional step in the search for enzymes with certain substrate specificities among hydrolases. By combining protein structure prediction tools like Alphafold^12^ with molecular docking of the substrate, such tests could also be conducted automatically without experimental protein structures. This allows further refinement of the search outcomes from bioinformatics before experimental testing: an approach that is analogous to virtual screening in drug design.

Finally, a key experimental observation in this work is that when *Ad*Cut was in the presence of both PET and PBS, its activity on PBS decreased. Substrate specificities are an important consideration for the engineering of enzymes in the large-scale biocatalytic recycling of plastic waste where blends of different polymers might be encountered. This work shows that in an industrial context, off-target binding could have an important impact on the activity of the enzymes and reduce their efficiency. Again, modeling may prove to be a valuable tool in predicting when this is likely to occur.

## Supporting Information Available

Particle size analysis; crystal structure of *Is*PETase and *Ad*Cut; nucleotide sequences; amino acid sequences; crystallographic data and refinement statistics; and R2 values for the fitting of a Langmuir adsorption (PDF)
