# Supplementary material for: Understanding the Catalytic Efficiency of Two Polyester Degrading Enzymes: An Experimental and Theoretical Investigation
Source: ACS Omega. 2024 Oct 23;9(44):44724–33. doi: 10.1021/acsomega.4c06528 (PMC11541480; doi:10.1021/acsomega.4c06528)
Supplement: Supplementary file 1 — ao4c06528_si_001.pdf [file ao4c06528_si_001.pdf]

Supplementary Information

Understanding the Catalytic Efficiency of Two Polyester Degrading Enzymes: An Experimental and Theoretical Investigation

Matilda Clark,[a] Konstantinos Tornesakis,[a] Gerhard König,[a,†] Michael Zahn,[a, ‡] Bruce R. Lichtenstein,[a] Andrew R. Pickford[a] and Paul A. Cox [a,b*]

[a] Centre for Enzyme Innovation, University of Portsmouth, St Michael's Building, Portsmouth, PO1 2DT, UK.

[b] School of Pharmacy and Biomedical Sciences, University of Portsmouth, St Michael's Building, Portsmouth, PO1 2DT, UK.

Corresponding author. E-mail: paul.cox@port.ac.uk

† *Current Address: Research Center for Pharmaceutical Engineering GmbH, 8010 Graz, Austria*

‡ *Current Address: Biozentrum, Martin-Luther-University Halle-Wittenberg, 06120 Halle, Germany*

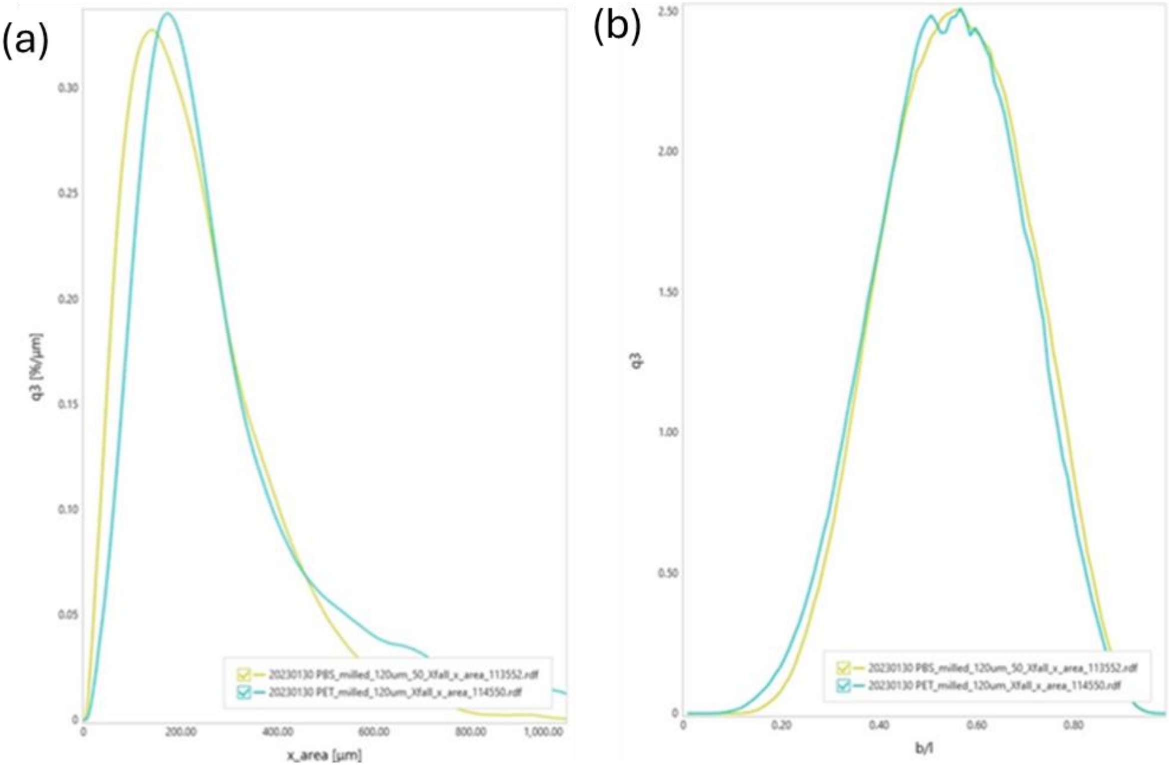

Figure S1 Particle size analysis. Population distributions are shown for (a) the cross-sectional area and (b) aspect ratio (breadth/length) as determined from dynamic image analysis on the cryomilled PET powder. In a, *x area* is the diameter of a circle within the equivalent cross-sectional area as the particle. The median *x area* of PBS is 208.03μm and the median *x area* of PET is 235.8μm.

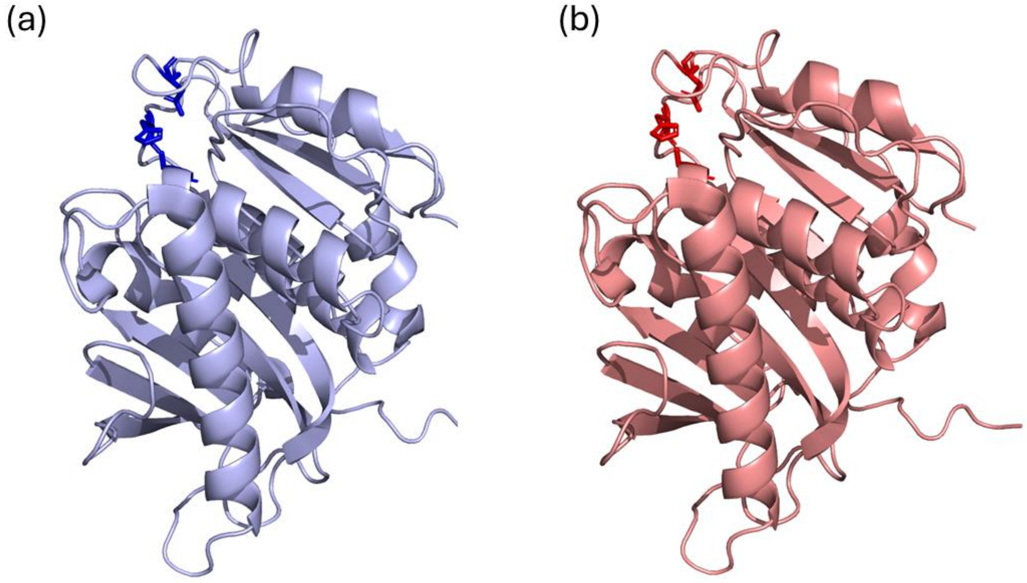

Figure S2 Crystal structure of *Is*PETase (a) (PDB:6EQE) and crystal structure of *Ad*Cut (b) (PDB:8C65). The catalytic triad of each enzyme is shown as sticks.

Table S1 Nucleotide sequence of the enzymes used in this study.

Enzyme name	Nucleotide Sequence of the Expressed Enzyme

*Is*PETase	ATG AAC TTC CCC CGT GCC TCG CGC CTT ATG CAG GCT GCT<br>GTG CTG GGC GGC CTT ATG GCC GTT TCC GCA GCG GCC ACC<br>GCG CAG ACC AAT CCG TAT GCG CGC GGC CCC AAC CCT ACC<br>GCC GCC TCG TTG GAA GCC AGC GCG GGA CCC TTT ACC GTT<br>CGT AGC TTT ACC GTT AGC CGT CCG TCC GGA TAT GGT GCA<br>GGG ACC GTC TAT TAC CCA ACC AAT GCA GGC GGC ACC GTT<br>GGC GCG ATT GCA ATC GTC CCC GGG TAC ACC GCG CGT CAA<br>AGC AGC ATT AAG TGG TGG GGT CCG CGC TTA GCT AGC CAT<br>GGC TTT GTG GTT ATT ACC ATC GAT ACG AAC AGC ACT CTA<br>GAC CAG CCC AGC AGC CGT AGC TCG CAA CAG ATG GCC GCG<br>CTT CGT CAA GTT GCG AGC TTG AAC GGG ACC AGC AGT AGC<br>CCG ATT TAC GGA AAG GTC GAT ACT GCC CGC ATG GGT GTG<br>ATG GGC TGG TCA ATG GGG GGC GGC GGT TCA CTT ATT AGC<br>GCC GCG AAC AAC CCG AGT TTA AAA GCA GCG GCA CCG CAG<br>GCG CCA TGG GAC TCT TCA ACC AAC TTC AGC AGT GTT ACC<br>GTG CCG ACG CTG ATT TTC GCG TGC GAG AAT GAT AGC ATT<br>GCA CCG GTG AAC AGC AGC GCG CTG CCG ATT TAT GAT AGC<br>ATG TCC CGC AAC GCA AAA CAG TTT CTG GAA ATT AAC GGC<br>GGT AGC CAC TCT TGT GCC AAC TCT GGG AAC AGC AAC CAG<br>GCA CTG ATC GGA AAA AAA GGG GTT GCA TGG ATG AAA CGA<br>TTC ATG GAT AAT GAC ACC CGT TAC TCA ACC TTC GCC TGT<br>GAG AAT CCC AAC AGC ACA CGC GTG TCG GAT TTT CGC ACC<br>GCG AAC TGT TCC CTC GAG CAC CAC CAT CAC CAC CAC TGA
*Ad*Cut	ATG CAC CTG CCG CGT TCC CGC TGG GAT ATC CCG TTC AAA<br>GAG GAA ACT ACC ATG ACC CAT CAT TTC TCT GTT CGC GCT<br>CTG CTG GCG GCC GGT GCT CTC CTG GCG TCC GCA GCA GTT<br>AGC GCA CAG ACC AAC CCG TAC GAG CGT GGT CCG GCC CCG<br>ACC ACC TCG TCT CTG GAA GCT TCT CGT GGT CCG TTC TCT<br>TAC CAA AGT TTT ACT GTC TCC CGC CCG TCT GGT TAC CGT<br>GCC GGG ACC GTG TAC TAT CCA ACT AAC GCG GGT GGC CCT<br>GTA GGC GCA ATT GCT ATC GTT CCG GGG TTC ACC GCA CGC<br>CAG AGC TCT ATT AAT TGG TGG GGC CCA CGT CTT GCA TCG<br>CAC GGT TTC GTT GTT ATC ACT ATC GAC ACT AAC TCT ACT<br>CTG GAT CAG CCA GAT AGC CGT TCC CGC CAA CAG ATG GCG<br>GCT CTG AGC CAG GTT GCA ACC TTA AGT CGT ACC AGC AGC<br>TCC CCG ATC TAC AAT AAA GTG GAC ACC TCC CGT CTG GGC<br>GTT ATG GGC TGG TCA ATG GGT GGC GGC GGT TCT CTG ATC<br>AGC GCC CGA AAC AAC CCG TCA ATT AAA GCG GCA GCG CCA<br>CAA GCC CCG TGG TCT GCA TCC AAG AAC TTT AGC TCA CTG<br>ACC GTG CCG ACC CTG ATC ATT GCC TGC GAA AAC GAC ACC<br>ATC GCA CCT GTG AAC CAG CAT GCT GAT ACT TTC TAC GAT<br>AGC ATG AGC CGT AAC CCG CGT GAA TTC CTG GAA ATT AAC<br>AAC GGT AGC CAC AGC TGC GCT AAT TCC GGC AAC TCA AAT<br>CAG GCG CTG CTG GGT AAA AAA GGG GTT GCT TGG ATG AAA<br>CGT TTC ATG GAT AAT GAT CGC CGT TAC ACC AGC TTC GCG<br>TGT TCC AAC CCG AAC TCT TAC AAC GTT AGC GAT TTC CGC<br>GTA GCG GCT TGC AAC CTC GAG CAC CAC CAC CAC CAC CAC<br>TGA

In blue: His-tag; in red: Signal peptide

Table S2 Amino Acid Sequence of the Enzymes Used in this Study.

Enzyme name	Amino Acid Sequence of the Expressed Enzyme
*Is*PETase	MNFPRASRLM QAAVLGGLMA VSAAATAQTN PYARGPNPTA ASLEASAGPF<br>TVRSFTVSRP SGYGAGTVYY PTNAGGTVGA IAIVPGYTAR QSSIKWWGPR<br>LASHGFVVIT IDTNSTLDQP SSRSSQQMAA LRQVASLNGT SSSPIYGKVD<br>TARMGVMGWS MGGGGSLISA ANNPSLKAAA PQAPWDSSTN FSSVTVPTLI<br>FACENDSIAP VNSSALPIYD SMSRNAKQFL EINGGSHSCA NSGNSNQALI<br>GKKGVAWMKR FMDNDTRYST FACENPNSTR VSDFRTANCS LEHHHHHH
*Ad*Cut	MHLPRSRWDI PFKEETTMTH HFSVRALLAA GALLASAAVS AQTNPYERGP<br>APTTSSLEAS RGPFSYQSFT VSRPSGYRAG TVYYPTNAGG PVGAIAIVPG<br>FTARQSSINW WGPRLASHGF VVITIDTNST LDQPDSRSRQ QMAALSQVAT<br>LSRTSSSPIY NKVDTSRLGV MGWSMGGGGS LISARNNPSI KAAAPQAPWS<br>ASKNFSSLTV PTLIIACEND TIAPVNQHAD TFYDSMSRNP REFLEINNGS<br>HSCANSGNSN QALLGKKGVA WMKRFMDNDR RYTSFACSNP NSYNVSDFRV<br>AACNLEHHHH HH

In blue: His-tag; in red: Signal peptide

Table S3 Crystallographic data and refinement statistics.

	*Ad*Cut
Data collection	
Beamline	DLS I03
Wavelength	0.9762
Space group	$P2_1$
Cell dimensions	
a, b, c (Å)	45.8, 96.4, 58.5
α, β, γ (°)	90.0, 111.0, 90.0
Resolution (Å)	54.63-1.49 (1.59-1.49) [a]
R_{merge} [%]	19.5 (126.2)
R_{pim} [%]	8.0 (50.8)
$\langle I/\sigma(I)\rangle$	7.1 (1.5)
Completeness (%)	78.1 (65.5) [b]
Redundancy	6.7 (7.0)
CC(1/2)	0.995 (0.529)
Refinement	
R_{work} / R_{free}	17.5 / 21.5
Ramachandran plot	
most favored	97.1
allowed [%]	2.9
disallowed [%]	0.0
No. atoms protein	4006
water	405
B-factors protein	12.1
water	19.7
R.m.s. deviations	
Bond lengths (Å)	0.0086
Bond angles (°)	1.52
pdb-code	8C65

[a] values in parentheses are for the highest-resolution shell [b] ellipsoidal completeness

Table S4: R^2 values for the fitting of a Langmuir adsorption isotherm to the data in Figure 4.

	*Ad*Cut	*Is*PETase
PBS	0.7529	0.8099
PET	0.7653	0.6256
